# Reply to Holm et al.: Careful training is a good practice

**DOI:** 10.1073/pnas.2305855120

**Published:** 2023-05-30

**Authors:** Rongguang Wang, Pratik Chaudhari, Christos Davatzikos

**Affiliations:** ^a^Department of Electrical and Systems Engineering, School of Engineering and Applied Science, University of Pennsylvania, Philadelphia, PA 19104; ^b^Center for Biomedical Image Computing and Analytics, Perelman School of Medicine, University of Pennsylvania, Philadelphia, PA 19104; ^c^Department of Computer and Information Science, School of Engineering and Applied Science, University of Pennsylvania, Philadelphia, PA 19104; ^d^Department of Radiology, Perelman School of Medicine, University of Pennsylvania, Philadelphia, PA 19104

We would like to thank Holm et al. (1) for beginning the discussion and qualifying the findings of our paper. We believe that the narrative in our manuscript is already very clear on the points raised by their letter.

## What Are Good Ways to Mitigate Bias?

In (2), we provide rigorous evidence that bias in machine learning models—as evaluated by multiple metrics such as i) area under the ROC curve (AUC), ii) demographic parity difference (DPD), iii) equalized odds difference (EOD), iv) equal opportunity difference (EO), v) predictive parity difference (PPD), and vi) generalized entropy index (GEI)—can be significantly mitigated using well-known safeguards against poor generalization, e.g., adequate preprocessing of the data and good hyperparameter tuning and model selection, for diagnosing Alzheimer’s disease, schizophrenia, and autism spectrum disorder using neuroimaging data. The narrative of the paper is precise in that it does not imply anything more than this statement. We do not claim that i) these techniques are sufficient to remove bias (title of the paper), ii) these metrics are the only appropriate ways to characterize bias in all situations (caption of Fig. 1), iii) our findings hold for other problems (title), or iv) bias can be eliminated completely using algorithmic procedures (first paragraph of discussion which refers to Larrazabal et al. 2020).

## How to Choose Metrics for Estimating Bias?

We agree with Holm et al. that choosing a metric to estimate bias is an ethical question and it depends on the context. It is hard to imagine that one metric, or even a few metrics, can fully characterize the performance of a system that interacts with different people in the society. This is why the title of our manuscript says that bias is mitigated—not eliminated. The question of how to choose appropriate metrics for estimating bias is beyond the scope of our manuscript.

In summary, we agree with the concluding statement of Holm et al.: “...we warn against establishing fixed recommended metrics for measuring fairness.” But our findings do suggest that researchers can benefit by using well-established techniques for data processing, hyperparameter tuning, and model selection in their efforts to mitigate bias.
